# Explicitly predicting outcomes enhances learning of expectancy-violating information

**DOI:** 10.3758/s13423-022-02124-x

**Published:** 2022-06-29

**Authors:** Garvin Brod, Andrea Greve, Dietsje Jolles, Maria Theobald, Elena M. Galeano-Keiner

**Affiliations:** 1 DIPF | Leibniz Institute for Research and Information in Education, Rostocker Str. 6, 60323 Frankfurt am Main, Germany; 2 Department of Psychology, Goethe University, Frankfurt, Germany; 3 MRC Cognition & Brain Sciences Unit, University of Cambridge, Cambridge, UK; 4 Institute of Education and Child Studies, Universiteit Leiden, Leiden, The Netherlands

**Keywords:** Violation of expectation, Prediction error, Active learning, Surprise, Pupillometry

## Abstract

Predictive coding models suggest that the brain constantly makes predictions about what will happen next based on past experiences. Learning is triggered by surprising events, i.e., a prediction error. Does it benefit learning when these predictions are made deliberately, so that an individual explicitly commits to an outcome before experiencing it? Across two experiments, we tested whether generating an explicit prediction before seeing numerical facts boosts learning of expectancy-violating information relative to doing so post hoc. Across both experiments, predicting boosted memory for highly unexpected outcomes, leading to a U-shaped relation between expectedness and memory. In the post hoc condition, memory performance decreased with increased unexpectedness. Pupillary data of Experiment 2 further indicated that the pupillary surprise response to highly expectancy-violating outcomes predicted successful learning of these outcomes. Together, these findings suggest that generating an explicit prediction increases learners’ stakes in the outcome, which particularly benefits learning of those outcomes that are different than expected.

**Supplementary Information:**

The online version contains supplementary material available at 10.3758/s13423-022-02124-x.

## Introduction

Our brain constantly generates predictions based on past experiences, according to predictive coding theories. Learning is triggered by surprising events, i.e., a prediction error (Bar, 2007; Friston, 2010; Henson & Gagnepain, 2010). This conjecture resonates well with formal mathematical conceptualizations of learning, such as in Bayesian statistics or information theory (Friston, 2018). In these theories, which primarily focus on nondeclarative learning, predictions are assumed to be generated implicitly (or unconsciously). Yet, humans can also deliberately engage in predicting, particularly in the context of declarative learning. This raises a series of questions: If our brains are constantly making predictions, does it have an added benefit when predictions are made deliberately? Does actively making a prediction affect the way in which unexpected information is processed? Does it affect how well unexpected information is later recalled?

In a quest to answer these questions, the current series of studies tested the hypothesis that generating an explicit prediction boosts later memory for expectancy-violating (i.e., incorrectly predicted) outcomes. We further assessed the role of surprise in this effect. More specifically, we tested whether memory for expectancy-violating outcomes is a function of how surprised a person was when encountering that information. Before elaborating on previous findings that give rise to these hypotheses, we briefly touch on the methodological issue of how best to conceptualize and measure surprise.

Psychology commonly defines surprise as an individual’s *emotional reaction* to a violation of expectations (Reisenzein, Horstmann, & Schützwohl, 2019), but the best way to conceptualize and measure surprise is still debated (Reisenzein et al., 2019). Here we conceptualize surprise as a highly dynamic process, i.e., the level of surprise experienced by the same (hypothetical) person about the same piece of expectancy-violating information varies from moment to moment, depending on the momentary strength of expectation for this specific piece of information relative to other information.

As a marker of surprise, we used the transient increase in pupil size that peaks approximately 1.5 s after an expectancy-violating outcome. We call it the pupillary surprise response (Brod et al., 2018) because it constitutes a reliable marker of the physiological component of surprise (Krüger, Bartels, & Krist, 2020; Preuschoff, ’t Hart, & Einhäuser, 2011; Reisenzein, Bördgen, Holtbernd, & Matz, 2006; Theobald & Brod, 2021). The increase in pupil size following expectancy-violations is driven by the release of norepinephrine in the brainstem’s locus coeruleus (Joshi, Li, Kalwani, & Gold, 2016; Lawson, Bisby, Nord, Burgess, & Rees, 2021). Although changes in pupil size do not directly track the *feeling of* surprise, they do reflect an objective moment-to-moment measure of the physiological underpinnings of surprise.

Recent findings in various age groups suggest pupillary surprise responses are enhanced when participants make explicit predictions before an outcome is presented (Breitwieser & Brod, 2021; Brod et al., 2020, 2018; Theobald & Brod, 2021; for a review, see Brod, 2021). Such explicit predictions have been found to lead to enhanced curiosity (Brod & Breitwieser, 2019), which is thought to reflect an increase in the subjective value of the outcome and in the momentary strength of expectation. When the outcome differs from the prediction, the perceived violation of expectation and surprise are increased (Brod & Breitwieser, 2019). Less clear, however, is whether and how this leads to better memory. The increased surprise response after explicit predictions suggests that explicit predictions increase attention to the correct result/feedback (Brod et al., 2018). Expectancy-violating outcomes are assumed to particularly benefit from this enhanced attention because they cannot be easily integrated into existing knowledge structures (Chinn & Brewer, 1993). Because this integration process is heavily dependent on top-down attentional control mechanisms mediated by the prefrontal cortex (Brod, Lindenberger, & Shing, 2017; Hartley, Nussenbaum, & Cohen, 2021), it is further unclear whether the memory benefit is subject to developmental changes.

Here we investigate these hypotheses across two experiments using behavioral and pupillary measures. The first experiment, performed in high-school students, confirmed our hypothesis that making explicit predictions before an outcome is presented improves memory for unexpected information compared to making explicit predictions after an outcome is presented. Experiment 2, performed in university students, replicated this behavioral benefit of explicit predictions and furthered our understanding of the underlying mechanism by showing pupillary responses to highly expectancy-violating events were predictive of subsequent memory.

## Experiment 1

Participants were asked to predict numerical trivia facts to test the hypothesis that generating an explicit prediction boosts later memory for expectancy-violating outcomes. We compared the explicit prediction condition to a post hoc evaluation condition (henceforth called postdiction condition). The postdiction condition allowed us to estimate the degree of expectancy-violation in the same way as in the explicit prediction condition and thus enabled us to compare the relation between memory performance and expectancy-violation in the same way as in the explicit prediction condition. We further hypothesized that, in the prediction condition, memory of an expectancy-violation is a function of the pupillary surprise response.

### Methods

#### Participants

We tested *n* = 24 high-school students (*M*_Age_ = 11.92 years, *SD*_Age_ = 1.25 [10, 14], 41.7 % female). We aimed to test 29 participants, following a previous study with the same number of trials per condition (Brod & Breitwieser, 2019). However, data acquisition was halted after 24 participants because of a Covid lockdown. The study is, thus, at risk of being underpowered (see *Discussion* section). Data from one participant had to be removed because of technical problems leading to the loss of eye-tracking data. The final sample consisted of *n* = 23 participants, which were recruited through partner schools. Participants gave written, informed consent prior to testing and received 10 Euro for participation. Ethics approval was obtained from the ethics committee of DIPF | Leibniz Institute for Research and Information in Education.

#### Design and stimuli

**Fig. 1:**
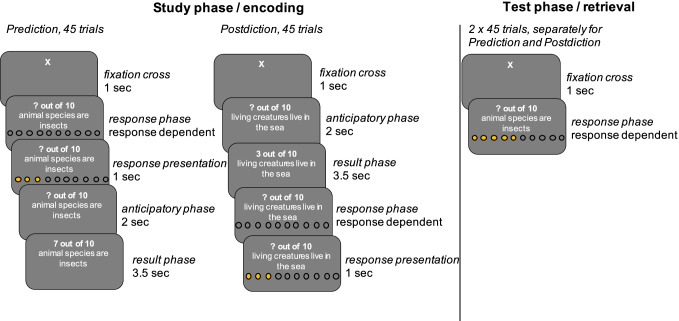
Schematic overview of the study and test phase of Experiment 1. The prediction and postdiction condition were performed in different blocks. In the study phase, participants predicted the numerical fact before seeing the correct number (prediction condition, 45 trials; left panel) or after seeing the correct number (postdiction condition, 45 trials; right panel). Each study phase was followed by a test phase, in which participants were asked to recall the correct number for each of the presented facts (order of facts was pseudorandomly reshuffled). White print against a gray background was used to reduce luminance contrasts

Stimuli comprised 90 numerical facts in the format of “X out of 10” (see Fig. 1), which is equivalent to a percentage estimate. Most of the facts were taken from the age-comparative study by Breitwieser and Brod (2021), which ensured that all facts were intelligible for both children and adults. We asked all participants right after the experiment whether they had known any of the facts beforehand, which was rarely the case in both age groups (i.e., median = 0, range 0–2). A key advantage of numerical facts is that they allow for a parametric analysis of the distance between the predicted outcome and the actual outcome (i.e., degree of expectancy-violation). In this fully within-subject experimental manipulation, participants had to state their prior expectancy either before seeing the correct number (prediction condition) or after seeing the correct number (postdiction condition). Prediction and postdiction conditions were performed in two different blocks with 45 facts each. The order of the blocks as well as the assignment of facts to blocks was counterbalanced across participants. Correct answers ranged between “1” and “9” (i.e., “0” and “10” were never correct).

#### Procedure

After a brief introduction into the computerized task and question format, participants began with either the prediction or postdiction block (counterbalanced). Each block consisted of a study phase followed by a test phase. The self-paced test phase was identical across blocks/condition and consisted of 45 facts presented during the respective study phase, in reshuffled order. The study phase differed between conditions in the order of slides (timings were identical) and the exact task during the “response phase” (also see Fig. 1). In the “response phase” of the prediction condition, participants had to predict the outcome (e.g., “? out of 10 animal species are insects”) by clicking on a 10-point visual analogue scale and were shown the correct number afterwards. The visual analogue scale was used to further aid intelligibility of the task for participants with low numeracy skills. In the postdiction condition, participants saw the correct number first and had to state afterwards – using the identical 10-point scale as in the prediction condition – what they would have predicted. In both conditions, participants were shown their response for 1 s (response presentation). The “anticipatory phase” was included to allow for a fair comparison of pupil dilation in the “result phase” between the prediction and postdiction condition (see next section). In sum, the procedure for the prediction and postdiction condition was highly similar, and total presentation time of the facts was identical.

After completion of the experiment, participants performed a brief task that assesses inter-individual differences in executive functions (Hearts & Flowers Task). Because of our focus on intra-individual effects, these data were not analyzed for the current article.

#### Stimulus presentation and eye-tracking procedures

The study phase was tailored to the measurement of changes in pupil size in response to the presentation of the correct number (i.e., during the “result phase”). Pupil size is strongly affected by changes in luminance. We therefore included a 2-s “anticipatory phase” prior to the result phases of each condition, which was visually identical to the “result phase” except the sentence showing the answer displayed an “X” instead of the correct number. This way the visual change from the anticipatory to the result phase was kept to an absolute minimum, preventing the need for the pupil to adapt to a new image in the result phase. It further ensured high comparability between the two conditions. In pilot experiments, we optimized the time interval of the “anticipatory phase” to ensure participants had enough time to read the full sentence especially in the postdiction condition, even though at times this might have led them to generate a prediction in the postdiction condition as well. Note, however, that if that was the case this should have reduced our condition difference (and, thus, work against our hypotheses).

Stimuli were presented using PsychoPy v1.83.02, which offers high timing precision. Participants were seated about 70 cm from a computer screen in a dimly lit room. The eye-tracking camera (EyeLink 1000, SR Research, Osgoode, Ontario, Canada) was placed below the computer screen and recorded at a frequency of 500 Hz throughout the experiment.

#### Data analyses

Data were analyzed using the statistical software package R (http://www.r-project.org). We performed logistic linear mixed-effects regression analyses using the *lme4* and *lmerTest* packages in order to test our purely intra-individual hypotheses. For logistic models as used in this study, the *lmerTest* package provides z-values (i.e., standardized coefficients), which can be directly converted to p-values and confidence intervals. A conventional alpha level of .05 was applied to all tests. The degree of expectancy-violation (i.e., absolute difference between expected and observed outcome) and the pupillary marker of surprise were centered on their respective person means. Hence, the respective effects are pure estimates of within-person effects. A further advantage of person-mean-centering is that both individual differences and condition differences in the average degree of expectancy-violations should not interfere with the detection of quadratic effects. This is critical because the postdiction condition is known to produce hindsight bias (Brod, Hasselhorn, & Bunge, 2018), which was the case in the current study as well (i.e., smaller average difference between expected and observed outcome in the postdiction condition [mean = 1.63, SD = 1.43] than in the prediction condition [mean = 2.36, SD = 1.75]).

#### Analysis of the behavioral data

Participants’ memory performance (i.e., retrieval success 0/1) was entered as a dependent variable. It was predicted by the degree of expectancy-violation (i.e., 0–9 difference between the expected and actual outcome), condition (prediction, postdiction), and their interaction (Model 1). In a follow-up analysis, we ran separate models for the prediction (Model 2a) and postdiction (Model 2b) condition and tested both linear and quadratic effects of expectancy-violation on retrieval success by including the absolute difference between expected and observed outcome (prediction error) and the quadratic prediction error (computed as the square of prediction error linear) as predictors into the models. Models included a random intercept for participant and a random slope parameter for the effect of expectancy-violation. Random effects were allowed to co-vary freely (i.e., unstructured G-matrix).

#### Analysis of the pupillary data

We used itrackR (https://github.com/jashubbard/itrackR) and self-developed analysis scripts to analyze the pupillary data. We merged the pupillary and behavioral data. Then, blinks were removed and the missing values were interpolated using cubic spline interpolation. Next, we derived markers of surprise in the pupillary data using the same time windows as Brod and Breitwieser (2019). To derive a marker of surprise in the pupillary data, we calculated for each trial the average percentage change in pupil diameter 0.5 through 2 s after the onset of the result phase relative to a pupil baseline phase (100 ms before onset of the result phase until – 200 ms after the onset of the result phase). Because the baseline phase is rather short and late, we confirmed that results are the same when the analyses are conducted with an earlier, longer baseline (500 ms before onset of the result phase).

Before analyzing condition differences, we conducted outlier analyses for the pupillometric markers of surprise. The analysis served to identify trials where the pupil dilation response deviated strongly from the rest of the distribution, i.e., more than 3 SDs from the average pupil dilation response. We identified 35 outlier trials (out of 2,070 trials in total) for the pupillometric marker of surprise, which were excluded from further analysis (exclusion of 1.7% of the data).

### Results

#### Is memory enhanced for expectancy-violating outcomes?

**Fig. 2:**
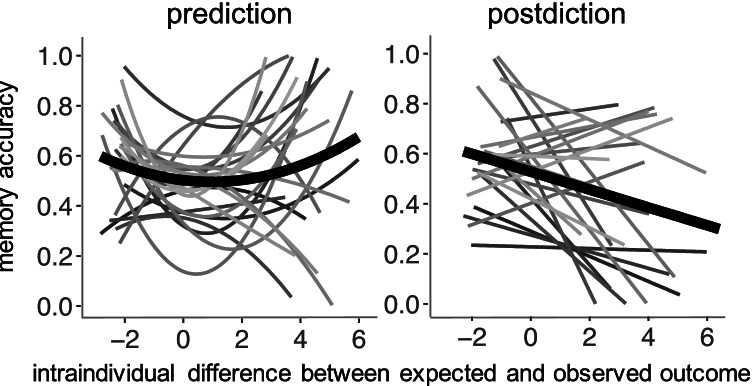
Relation between expectancy-violation (i.e., difference between expected and observed outcome; centered at the person mean) and memory accuracy, separately for the prediction (left panel) and postdiction (right panel) condition. The light grey lines show the best-fitting regression lines for each participant, the bold black line shows the best-fitting regression line at the group level. Memory performance followed a U-shaped function in the prediction condition: accuracy was highest for both highly expected and highly unexpected outcomes. By contrast, in the postdiction condition, memory performance linearly decreased the more unexpected the outcomes were

We observed no significant effect of condition (b = -0.090, SE = 0.092, p = .330) but a significant linear within-person effect of expectancy-violation on memory (b = -0.198, SE = 0.051, p < .001), which was qualified by a significant interaction with condition (b = 0.195, SE = 0.062, p = .002). As can be seen in Fig. 2, in the prediction condition, memory performance followed a U-shaped function of expectancy – being highest for both highly expectancy-consistent and highly expectancy-violating outcomes. In contrast, in the postdiction condition, memory performance continuously decreased with increasing unexpectedness of the outcome. We followed up on this pattern of results by also testing for an interaction between condition and a quadratic effect of expectancy-violation. Results indicated a significant effect of condition (b = -0.239, SE = 0.107, p = .026), no quadratic effect of expectancy-violation (b = -0.027, SE = 0.017, p = .121), but again a significant interaction (b = 0.061, SE = 0.024, p = .011).

We then tested both linear and quadratic effects separately for the two conditions. In the prediction condition, we observed a significant quadratic effect (b = 0.044, SE = 0.018, p = .012) but no linear effect (b = -0.054, SE = 0.043, p = .212) of degree of expectancy-violation. Model comparison showed that a model with both a linear and a quadratic parameter fitted the data better than a model that only included a linear effect (AIC_m1_= 1396.5 < AIC_m2_ = 1401.0, Chisq = 6.4562, df = 1, p = .011). In the postdiction condition, we observed a significant linear effect (b = -0.254, SE = 0.065, p < .001) but no quadratic effect (b = 0.027, SE = 0.022, p = .223). Model comparison showed that a model with both a linear and a quadratic parameter fitted the data better than a model that only included a quadratic effect (AIC_m1_ = 1355.8 < AIC_m2_ = 1368.8, Chisq = 14.993, df = 1, p < .001). In sum, these results show that memory is enhanced for both highly expectancy-violating and highly expectancy-consistent outcomes in the prediction condition (i.e., U-shape). In contrast, memory enhancements in the postdiction condition are limited to expectancy-consistent outcomes.

#### Does the pupillary surprise response predict learning?

To test our hypothesis that pupillary surprise in response to expectancy-violating outcomes is predictive of later memory, we added the baseline-corrected change in pupil size during the “result phase” to Model 1 as described for the behavioral data. In contrast to our hypothesis, we did not observe any significant effects involving the factor pupil size (see Table 1a). Thus, changes in pupil size during the “result phase” were not predictive of later memory.

**Table 1:** Logistic linear mixed regression models – predicting memory by expectancy-violation, pupil size, and condition

Fixed effects	a) Experiment 1			b) Experiment 2		
	*Estimate*	*SE*	*p-value*	*Estimate*	*SE*	*p-value*
(Intercept)	0.163	0.121	0.18	0.405	0.080	< 0.001
Condition	-0.097	0.092	0.294	/	/	/
Expectancy-Violation	-0.196	0.051	**< **0.001	-0.032	0.026	0.229
Pupil Size	0.839	2.144	0.695	1.812	1.678	0.28
Condition*Expectancy-Violation	0.192	0.063	0.002	/	/	**/**
Condition*Pupil Size	-0.266	2.843	0.925	/	/	/
Expectancy-Violation*Pupil Size	1.567	1.647	0.341	2.186	0.986	0.027
Condition * Expectancy-Violation*Pupil Size	-2.031	2.031	0.311	/	/	/
Random effects (Variances)						
Intercept	0.24			0.132		
Prediction Error	0.002			0.00004		
Pupil Size	9.278			0.196		
N	23			28		
Observations	2035			2480		

### Discussion

Experiment 1 shows that generating an explicit prediction before encountering an outcome enhances learning of highly expectancy-violating information relative to doing so post hoc. This selective memory enhancement resulted in a U-shaped relation between expectancy and memory in the explicit prediction condition. In contrast, in the postdiction condition, memory performance continuously decreased with increasing unexpectedness of the outcome. The observed U-shape is in line with recent findings that suggest that – under ideal circumstances – both highly expectancy-consistent and highly expectancy-violating events are better remembered than expectancy-neutral events (Greve, Cooper, Tibon, & Henson, 2019; Quent, Henson, & Greve, 2021). One interesting question is whether our observed patterns of results generalize to other populations than the high-school students tested here. In a small behavioral age-comparative study with both sixth-grade students and adults, we found a highly similar pattern of results, and no indication of an interaction with age (see Online Supplementary Material). These findings provide some corroborating evidence for the robustness of the reported effect that generating an explicit prediction enhances memory for highly expectancy-violating information.

However, Experiment 1 failed to provide evidence that the pupillary surprise response was linked to later memory for expectancy-violating information in the prediction condition. This absence of evidence either suggests a different mechanism is at play or that our design, which was tailored to detect condition differences in prediction and postdiction, was insufficiently powered to detect pupillary surprise responses. This is addressed in Experiment 2.

## Experiment 2

Experiment 2 used the same numerical trivia facts questions as Experiment 1 but instead of presenting both learning conditions (i.e., prediction and postdiction), we focused on testing the prediction condition in both blocks, doubling the number of trials compared to Experiment 1.

### Methods

#### Participants

We tested 29 university students (*M*_Age_ = 23.07 years, *SD*_Age_ = 3.07, [19; 30], 69% female) to obtain a target sample size of *n* = 28. Sample size was determined a priori using G*Power with the following settings (based on Experiment 2 in Brod et al., 2018): 2 × 2 repeated-measures ANOVA, effect size *f* = .25, *alpha* = .05, *beta* = .90, correlation among repeated measures = .7. However, we decided after the data had been collected (but before performing any analyses) that it is more appropriate to perform the analyses using linear mixed models because the number of highly expectancy-violating trials varies across participants. A power simulation for linear mixed-effect models^1^ indicated a power of 81.10% (confidence interval: 71.93, 88.16) for a two-way interaction effect. Data from one participant were discarded due to inadequate use of the confidence scale, i.e., the participant always indicated the same level of confidence across all trials. Participants were recruited through bulletins at a large university campus. They gave written, informed consent prior to testing and received 10 Euro for participation. Ethics approval was obtained from the ethics committee of DIPF | Leibniz Institute for Research and Information in Education.

#### Design and stimuli

Stimuli were identical to Experiment 1. The explicit prediction task was performed in two blocks with 45 facts each. The order of the blocks as well as the assignment of facts to blocks was counterbalanced across participants. Correct answers ranged between “1” and “9” meaning that “0” and “10” were never correct.

#### Procedure

The procedure was the same as for the prediction condition of Experiment 1, in addition, participants provided confidence ratings after having generated a prediction. They were asked to rate their confidence in the prediction on a scale from 1 (“not confident”) to 5 (“very confident”). After the self-paced rating, participants saw the initial question again (anticipation phase; 2 s) followed by the presentation of the correct number (results phase, 4 s). These confidence ratings were included with a view to explore whether confidence interacts with expectancy in predicting successful memory formation.

#### Data analyses

Preprocessing procedures and time windows for the pupillary data were identical to Experiment 1.

### Results

#### Is memory enhanced for expectancy-violating outcomes?

**Fig. 3:**
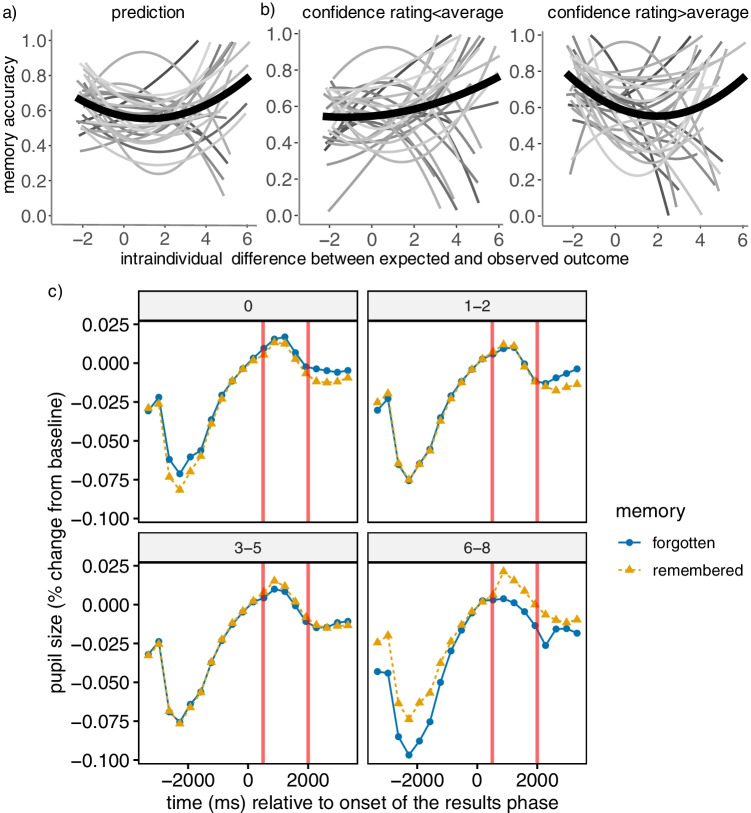
(**a**) Relation between expectancy-violation (i.e., difference between expected and observed outcome; centered at the person mean) and memory accuracy in the prediction condition. The light grey lines show the best-fitting regression lines for each participant, the bold black line shows the best-fitting regression line at the group level. (**b**) Interaction between expectancy-violation and memory, separately for lower (left panel) and higher (right panel) than individual mean confidence rating. (**c**) Time course of mean pupil dilation across a study trial as a function of memory (=recall success 0/1) and the degree of expectancy-violation/absolute difference between expected and observed outcomes (collapsed into four bins): 0 = no expectancy violation, 1–2 = small expectancy-violation, 3–5 = medium expectancy-violation, 6–8 = high expectancy-violation). Red vertical lines correspond to the analysis window used for measuring the pupillary surprise response (i.e., “result phase”)

To test the hypothesized U-shaped relation between expectancy and memory, as in Experiment 1, we performed a logistic linear mixed-effects regression in which retrieval success (0/1) was predicted by both linear and quadratic effects of person-mean-centered degree of expectancy-violation. This time, both the linear (*b* = -0.070, *SE* = 0.032, *p* = .028) and the quadratic effect (*b* = 0.028, *SE* = 0.013, *p* = .029) reached significance, likely due to higher statistical power. Model comparison showed that a model with both a linear and a quadratic parameter fitted the data better than a model that only included a linear effect (*AIC*_*m1*_ = 3312.9 < *AIC*_*m2*_ = 3315.7, *Chisq* = 4.806, *df* = 1, *p* = .028). As can be seen in Fig. 3a, the relation between the degree of expectancy-violation and later memory is best captured as a U-shape, corroborating the results of Experiment 1. We also tested whether subjective confidence in the explicit prediction interacted with expectancy in predicting memory. Results of a logistic linear mixed-effects regression revealed a significant within-person effect of confidence (*b* = 0.190, *SE* = 0.050, *p* < .001), showing that higher confidence was associated with more successful recall within individuals. Further, a significant interaction between confidence and expectancy-violation (*b* = -0.140, *SE* = 0.030, *p* < .001) suggested that the U-shaped relation between expectancy and memory was stronger for predictions made with higher-than-average confidence (see Fig. 3b).

### Does the pupillary surprise response predict learning?

We tested whether retrieval success (0/1) was linked to expectancy-violation and changes in pupil size during the “result phase.” In line with our hypothesis, we found a significant expectancy × pupil size interaction (*b* = 2.186, *SE* = 0.986, *p* = .027; see Table 1b). As can be seen in Fig. 3c, this interaction was driven by the highly expectancy-violating outcomes (i.e., 6–8 difference between the predicted and observed number), for which a greater pupil size was associated with a higher likelihood of successful learning. This was not the case for expected or moderately expectancy-violating outcomes. Put differently, trials with the same level of discrepancy between expected and observed outcomes (i.e., bin 6–8) but greater pupillary surprise response were more likely to be remembered.

### Discussion

Experiment 2 replicates the U-shaped relation between expectancy and memory observed in the explicit prediction condition of Experiment 1, i.e., memory is best for both highly expectancy-consistent and highly expectancy-violating outcomes. Interestingly, this U-shape is particularly evident for predictions made with higher confidence. The pupillary surprise response for expectancy-violating outcomes was predictive of subsequent memory, confirming preferential encoding of those outcomes that were particularly surprising. Together, these findings suggest that generating an explicit prediction increases learners’ stakes in the outcome, which particularly benefits learning of those outcomes that are different than expected.

We have argued that the null effect from Experiment 1 could be the result of the low number of trials. To substantiate this speculation, we reran the pupillary analysis of Experiment 2 using a random subsample of n = 45 trials per participant (i.e., with the same number of trials as in Experiment 1). We repeated this analysis 1,000 times. The number of significant (*p* < .05) interaction effects between pupil size and expectancy violation was counted. Then, the p-value was calculated by dividing this number by 1000, and subsequently evaluated at a *p* < .05. Results revealed that 260 out of 1,000 analyses showed significant interaction effects, which corresponds to a p-value of *p* = .26. Thus, when using this lower number of trials, no significant pupil-behavior interaction was found in Experiment 2. These findings suggest that differences in power indeed underlay the differences in pupillary results between Experiments 1 and 2.

## General discussion

Here we provide compelling evidence that generating an explicit prediction boosts memory for expectancy-violating outcomes. The extent to which generating predictions benefits learning of expectancy-violating information is a function of how surprised people are by the outcome. We use pupillary responses as an objective measure of physiological surprise to gain a deeper understanding of what supports the mnemonic advantage of expectancy-violations. We show that pupillary responses are predictive of subsequent memory performance for highly expectancy-violating outcomes only, suggesting that they are preferentially encoded after explicit predictions.

The assumed neurophysiological mechanism driving the pupillary response is the release of norepinephrine in the locus coeruleus (Joshi, Li, Kalwani, & Gold, 2016; Lawson, Bisby, Nord, Burgess, & Rees, 2021). Theoretical models posit that norepinephrine leads to an upregulation of the sensitivity in cortical processing, which facilitates learning of information that is currently considered important (for detailed accounts, see Aston-Jones & Cohen, 2005; Mather, Clewett, Sakaki, & Harley, 2016; Sakaki, Ueno, Ponzio, Harley, & Mather, 2019). In line with these theoretical models, empirical evidence from a combined pupillometry and functional magnetic resonance imaging (fMRI) study suggests that locus coeruleus activity promotes memory encoding processes in the hippocampus (Clewett, Huang, Velasco, Lee, & Mather, 2018). Hippocampal involvement is considered to be particularly important for successful memory of expectancy-violating outcomes (Bein, Duncan, & Davachi, 2020; Lisman, Grace, & Duzel, 2011). These findings resonate well with a neuroscientific model (Van Kesteren, Ruiter, Fernández, & Henson, 2012), which proposes that memory is a U-shaped function of schema-congruency and that different brain systems support memory for the two ends of the U-shape: memory for expectancy-violating events are thought to be supported by the medial temporal lobes (MTL), whereas memory for expectancy congruent events is hypothesized to be supported by the neocortex, for which the medial prefrontal cortex (mPFC) plays a key role.

Our study is not the first to use pupillary surprise responses to determine their predictive role in learning (e.g., Kafkas, 2021; Madore et al., 2020). One recent study (Kafkas, 2021) found that objects that violated a previously learned rule are better remembered when associated with an increase in pupil size, while objects that are consistent with a rule were better remembered when linked to a decrease in pupil size. In that study, participants first trained symbol-stimulus associations before “correct” or “incorrect” symbol-stimulus sequences were presented at encoding. Therefore, even though participants did not generate an explicit prediction, they likely had strong prior expectations that were either confirmed or violated during encoding. Combining these results with ours, we speculate that increases in pupil size will be predictive of subsequent memory for expectancy-violating outcomes for which participants had strong prior expectations. When prior expectations aren’t strong already, they can be boosted by asking participants to generate an explicit prediction. Some support for the role of expectancy strength in learning of expectancy-violating outcomes can be drawn from a recent study that found memory enhancements for expectancy-violating outcomes only when participants later remembered their prediction (i.e., “strong predictions”; Bein, Plotkin, & Davachi, 2021). Future studies are clearly needed though to further qualify the relationship between active predictions, pupil responses, and subsequent memory performance.

Although we replicated the behavioral findings of the U-shaped function both within and across different age groups, our pupil dilation responses clearly differed across the two experiments. We have shown that the null effect from Experiment 1 was likely the result of the low number of trials, which was doubled in Experiment 2. It is possible, however, that an even higher number of trials in Experiment 2 might have shown predictive pupillary responses also in the postdiction condition. Akin to previous studies with this paradigm, the average difference between expected and observed outcome was smaller in the postdiction condition than in the prediction condition, whereas the variance of the differences was similar between conditions. This pattern suggests that highly expectancy-violating trials in the postdiction condition were *extremely* expectancy-violating for participants. Nevertheless, we did not find an association between pupil size and subsequent memory in the postdiction condition even for those trials (see Fig. 3c). Future studies with considerably more trials in the postdiction condition too could clarify the relation between expectancy-violation, pupil dilation, and memory for this condition as well.

In conclusion, although our brains may be constantly making (implicit) predictions, our data suggest that explicit predictions enhance learning of unexpected outcomes. Generating an explicit prediction before seeing a numerical fact boosted learning of expectancy-violating information in both adolescents and younger adults. While this effect should be replicated in younger age groups, our findings suggest that this effect could be universal and, thereby, age-invariant. Explicit predictions might increase the subjective value of the outcome, resulting in a stronger surprise response in case of expectancy violations, which in turn triggers increased attention to the outcome. Requiring learners to deliberately engage their model of the world thus helps to show them that, from time to time, they are still wrong.

## Supplementary Information

ESM 1 (DOCX 119 kb)

## Footnotes

1 The power simulation was conducted using the “simr” package in R and used a simulated data set based on the current sample and paradigm: 28 participants, 90 trials, fixed effect for interaction term = .05, value for random intercept = .01, value for variance in random intercept = .0002, critical value = 2, 1000 simulation. These input values were derived post hoc based on the results from a mixed-effects regression that tested the interaction between confidence and prediction error as a predictor of the pupillary surprise response.

## References

[CR1] Aston-Jones G, Cohen JD (2005). An integrative theory of locus coeruleus-norepinephrine function: adaptive gain and optimal performance. Annual Review of Neuroscience.

[CR2] Bar M (2007). The proactive brain: using analogies and associations to generate predictions. Trends in Cognitive Sciences.

[CR3] Bein O, Duncan K, Davachi L (2020). Mnemonic prediction errors bias hippocampal states. Nature Communications.

[CR4] Bein O, Plotkin NA, Davachi L (2021). Mnemonic prediction errors promote detailed memories. Learning & Memory.

[CR5] Breitwieser J, Brod G (2021). Cognitive prerequisites for generative learning: Why some learning strategies are more effective than others. Child Development.

[CR6] Brod, G. (2021). Predicting as a learning strategy. *Psychonomic Bulletin and Review, 28, 1839–1847.* 10.3758/s13423-021-01904-1 PMC8642250 33768503

[CR7] Brod G, Breitwieser J (2019). Lighting the wick in the candle of learning: generating a prediction stimulates curiosity. Npj Science of Learning.

[CR8] Brod G, Lindenberger U, Shing YL (2017). Neural activation patterns during retrieval of schema-related memories: differences and commonalities between children and adults. Developmental Science.

[CR9] Brod G, Hasselhorn M, Bunge SA (2018). When generating a prediction boosts learning: The element of surprise. Learning and Instruction.

[CR10] Brod G, Breitwieser J, Hasselhorn M, Bunge SA (2020). Being proven wrong elicits learning in children – but only in those with higher executive function skills. Developmental Science.

[CR11] Chinn CA, Brewer WF (1993). The Role of Anomalous Data in Knowledge Acquisition: A Theoretical Framework and Implications for Science Instruction. Review of Educational Research.

[CR12] Clewett DV, Huang R, Velasco R, Lee TH, Mather M (2018). Locus coeruleus activity strengthens prioritized memories under arousal. Journal of Neuroscience.

[CR13] Friston K (2010). The free-energy principle: A unified brain theory?. Nature Reviews Neuroscience.

[CR14] Friston K (2018). Does predictive coding have a future?. Nature Neuroscience.

[CR15] Greve A, Cooper E, Tibon R, Henson RN (2019). Knowledge Is Power: Prior Knowledge Aids Memory for Both Congruent and Incongruent Events , but in Different Ways. Journal of Experimental Psychology: General.

[CR16] Hartley CA, Nussenbaum K, Cohen AO (2021). Interactive Development of Adaptive Learning and Memory. Annual Review of Developmental Psychology.

[CR17] Henson RN, Gagnepain P (2010). Predictive, interactive multiple memory systems. Hippocampus.

[CR18] Joshi S, Li Y, Kalwani RM, Gold JI (2016). Relationships between Pupil Diameter and Neuronal Activity in the Locus Coeruleus, Colliculi, and Cingulate Cortex. Neuron.

[CR19] Kafkas A (2021). Encoding-linked pupil response is modulated by expected and unexpected novelty: Implications for memory formation and neurotransmission. Neurobiology of Learning and Memory.

[CR20] Krüger M, Bartels W, Krist H (2020). Illuminating the dark ages: Pupil dilation as a measure of expectancy violation across the life span. Child Development.

[CR21] Lawson RP, Bisby J, Nord CL, Burgess N, Rees G (2021). The Computational, Pharmacological, and Physiological Determinants of Sensory Learning under Uncertainty. Current Biology.

[CR22] Lisman J, Grace AA, Duzel E (2011). A neoHebbian framework for episodic memory; role of dopamine-dependent late LTP. Trends in Neurosciences.

[CR23] Madore KP, Khazenzon AM, Backes CW, Jiang J, Uncapher MR, Norcia AM, Wagner AD (2020). Memory failure predicted by attention lapsing and media multitasking. Nature.

[CR24] Mather M, Clewett D, Sakaki M, Harley CW (2016). Norepinephrine ignites local hotspots of neuronal excitation: How arousal amplifies selectivity in perception and memory. Behavioral and Brain Sciences.

[CR25] Preuschoff K, ’t Hart BM, Einhäuser W (2011). Pupil dilation signals surprise: Evidence for noradrenaline’s role in decision making. Frontiers in Neuroscience.

[CR26] Quent JA, Henson RN, Greve A (2021). A predictive account of how novelty influences declarative memory. Neurobiology of Learning and Memory.

[CR27] Reisenzein R, Bördgen S, Holtbernd T, Matz D (2006). Evidence for strong dissociation between emotion and facial displays: The case of surprise. Journal of Personality and Social Psychology.

[CR28] Reisenzein R, Horstmann G, Schützwohl A (2019). The Cognitive-Evolutionary Model of Surprise: A Review of the Evidence. Topics in Cognitive Science.

[CR29] Sakaki M, Ueno T, Ponzio A, Harley CW, Mather M (2019). Emotional arousal amplifies competitions across goal-relevant representation: A neurocomputational framework. Cognition.

[CR30] Theobald, M., & Brod, G. (2021). Tackling scientific misconceptions: The element of surprise. *Child Development, 92*(5), 2128–2141. 10.1111/cdev.13582 33969879

[CR31] Van Kesteren MTR, Ruiter DJ, Fernández G, Henson RN (2012). How schema and novelty augment memory formation. Trends in Neurosciences.

