# Supplementary material for: Explicitly predicting outcomes enhances learning of expectancy-violating information
Source: Psychon Bull Rev. 2022 Jun 29;29(6):2192–201. doi: 10.3758/s13423-022-02124-x (PMC9722848; doi:10.3758/s13423-022-02124-x)
Supplement: Supplementary file 1 — (DOCX 119 kb) [file 13423_2022_2124_MOESM1_ESM.docx]

**Appendix**

To test whether the differences in memory performance between the prediction and postdiction condition observed in Experiment 1 are robust and generalize to older age groups, Experiment 1 was repeated in a mixed-age group of Dutch students.

***Participants***

We tested *n* = 18 adults (*M*_Age_ = 23.44, *SD*_Age_ = 2.97, 77.8 % female) and *n* = 19 sixth grade students (*M*_Age_ = 11.37, *SD*_Age_ = 0.50, 52.6 % female). Task data from one student were lost due to technical issues. In addition, we excluded data from an additional three sixth grade students because they misunderstood the task instructions for the postdiction condition (i.e., they repeated back the correct number instead of stating what they would have predicted), resulting in a sample of 15 sixth graders. Students and their parents (in case of sixth graders) gave written, informed consent prior to testing. Ethics approval was obtained from the ethics committee of the Institute of Education and Child Studies at Leiden University.

## Design & Stimuli

Design and stimuli were identical to Experiment 1 with the following exceptions: 1) Stimuli were presented in Dutch. Roughly one third of the items were direct translations of the German items. Other items were modified or entirely new in order to be appropriate for Dutch speakers. As in the main study, piloting was done to ensure that the facts were intelligible for children. 2) Prediction and postdiction conditions were performed in different blocks with 35 facts each. The order of blocks, as well as the assignment of facts to blocks, was counterbalanced across participants. 3) During the prediction condition, participants first made a prediction about the numerical fact by clicking on a 9-point scale (with no time-limit). Next, the correct number was displayed for 4.5 seconds. In the postdiction condition, participants first saw the correct number, and then had to state their own prediction. As in the main experiment, there was an ‘anticipatory phase’ of 1 second before the ‘results phase’ in both conditions. Between trials a fixation cross was presented for 1 second. 4) The test phase included all 70 numerical facts, with facts from both conditions randomly interspersed. Between trials a fixation cross was presented for 1 second.

## Procedure

Procedures were identical to Experiment 1 with the following exceptions: 1) Due to the ongoing COVID-19 pandemic, adults were tested online in a one-on-one Microsoft Teams session. The task was programmed in PsychoPy v2020.2.10 and ran online through Pavlovia.org. 2) Sixth grade students were tested in a quiet location in their school, in groups of two or three students at a time. Using laptops provided by the school, students performed the exact same online tasks as the adults. 3) Between the study phase and the test phase, all participants performed a 28-item questionnaire, including the 13-item Amsterdam Executive Function Inventory (Van der Elst et al., 2011), 5 questions about critical thinking, based on the Critical Thinking subtest of the Motivated Strategies for Learning Questionnaire (Pintrich et al., 1993), and 10 questions about curiosity and thrill seeking, based on the Joyous Exploration and Thrill-Seeking subtests of the Five-Dimensional Curiosity Scale (Kashdan et al., 2018). The questionnaire was delivered through Qualtrics. 4) No eye-tracking / pupillometry was performed. Questionnaire data were not analyzed for the current manuscript because we focused on intra-individual relations.

## Results

## As in Experiment 1, we performed a logistic linear mixed effects regression in which retrieval success (0/1) was predicted by the linear person-mean-centered degree of expectancy-violation. Age group was entered as an additional predictor to the model. We observed significant effects of age group (b = -0.647, SE = 0.188, p < .001), condition (b = -0.261, SE = 0.116, p = .025), and degree of expectancy-violation on memory (b = -0.218, SE = 0.067, p = .001). More importantly, we observed a significant interaction between condition and expectancy-violation on memory (b = 0.279, SE = 0.082, p < .001), and no interactions with age (all ps > .4). As can be seen in Figure S1, and in line with results of Experiment 1, memory performance was best described as a U-shape function of expectancy in the prediction condition (linear effect: b = .001, SE = 0.043, p = .972; quadratic effect: b = .031, SE = 0.017, p = .057; model comparison: AIC_m1_ = 1569.1 < AIC_m2_ = 1570.7, p = .055). This quadratic effect on memory did not interact with age (b = 0.022, SE = 0.027, p = .420). In the postdiction condition, memory performance continuously decreased with increasing unexpectedness of the outcome (linear effect: b = -.214, SE = 0.064, p < .001; quadratic effect: b = -.026, SE = 0.024, p = .291). This linear effect on memory did not interact with age either (b = -0.028, SE = 0.108, p = .799). In sum, results indicate that the findings of Experiment 1 are robust and generalizable to older students.

Figure S1. Relation between expectancy-violation (person-mean-centered) and memory accuracy, separately for the prediction (left panel) and postdiction (right panel) condition. The light grey lines show the best-fitting regression lines for each participant (n = 33 in total, collapsed across age groups), the bold black line shows the best-fitting regression line at the group level.

**References Appendix**

van der Elst, W., Ouwehand, C., van der Werf, G., Kuyper, H., Lee, N. C., & Jolles, J. (2011). The Amsterdam Executive Function Inventory (AEFI): psychometric properties and demographically-corrected normative data for adolescents aged between 15 and 18 years. *Journal of Clinical and Experimental Neuropsychology*, *33*(1), 1-12.

Kashdan, T. B., Stiksma, M. C., Disabato, D. J., McKnight, P. E., Bekier, J., Kaji, J., & Lazarus, R. (2018). The five-dimensional curiosity scale: Capturing the bandwidth of curiosity and identifying four unique subgroups of curious people*. Journal of Research in Personality*, *73*, 130–149.

Pintrich, P.R., Smith, D., Garcia, T., and McKeachie, W. (1991). *A Manual for the Use of the Motivated Strategies for Learning Questionnaire* (MSLQ), The University of Michigan, Ann Arbor, MI.
